# Incidental germline findings during comprehensive genomic profiling of pancreatic and colorectal cancer: single-centre, molecular tumour board experience

**DOI:** 10.1093/mutage/geae014

**Published:** 2024-05-22

**Authors:** Michal Eid, Jakub Trizuljak, Renata Taslerova, Martin Gryc, Jakub Vlazny, Sara Vilmanova, Martina Jelinkova, Alena Homolova, Stepan Tucek, Jan Hlavsa, Tomas Grolich, Zdenek Kala, Zdenek Kral, Ondrej Slaby

**Affiliations:** Department of Internal Medicine, Hematology and Oncology, University Hospital Brno, Faculty of Medicine, Masaryk University, Brno, Czech Republic; Department of Internal Medicine, Hematology and Oncology, University Hospital Brno, Faculty of Medicine, Masaryk University, Brno, Czech Republic; Department of Pathology, University Hospital Brno, Faculty of Medicine, Masaryk University, Brno, Czech Republic; Department of Internal Medicine, Hematology and Oncology, University Hospital Brno, Faculty of Medicine, Masaryk University, Brno, Czech Republic; Department of Pathology, University Hospital Brno, Faculty of Medicine, Masaryk University, Brno, Czech Republic; Department of Pathology, University Hospital Brno, Faculty of Medicine, Masaryk University, Brno, Czech Republic; Department of Pathology, University Hospital Brno, Faculty of Medicine, Masaryk University, Brno, Czech Republic; Department of Pathology, University Hospital Brno, Faculty of Medicine, Masaryk University, Brno, Czech Republic; Department of Internal Medicine, Hematology and Oncology, University Hospital Brno, Faculty of Medicine, Masaryk University, Brno, Czech Republic; Department of Surgery, University Hospital Brno, Faculty of Medicine, Masaryk University, Brno, Czech Republic; Department of Surgery, University Hospital Brno, Faculty of Medicine, Masaryk University, Brno, Czech Republic; Department of Surgery, University Hospital Brno, Faculty of Medicine, Masaryk University, Brno, Czech Republic; Department of Internal Medicine, Hematology and Oncology, University Hospital Brno, Faculty of Medicine, Masaryk University, Brno, Czech Republic; Department of Pathology, University Hospital Brno, Faculty of Medicine, Masaryk University, Brno, Czech Republic; Department of Molecular Medicine, Central European Institute of Technology, Masaryk University, Brno, Czech Republic; Department of Biology, Faculty of Medicine, Masaryk University, Brno, Czech Republic

**Keywords:** next-generation sequencing, pancreatic cancer, colorectal cancer, germline variants, somatic variants

## Abstract

Multidisciplinary molecular tumor boards (MTB) are already well established in many comprehensive cancer centers and play an important role in the individual treatment planning for cancer patients. Comprehensive genomic profiling of tumor tissue based on next-generation sequencing is currently performed for diagnostic and mainly predictive testing. If somatic genomic variants are identified, which are suspected to be pathogenic germline variants (PGVs), MTB propose genetic counseling and germline DNA testing. Commonly used comprehensive genomic profiling approaches of tumor tissue do not include a matched germline DNA control. Therefore, the detection of PGVs could be only predicted based on the content of tumor cells (CTC) in selected tumor area (%) and variant allele frequency score (%). For conclusion, the role of a medical geneticist is essential in these cases. The overall prevalence of PGVs in patients with pancreatic ductal adenocarcinoma (PDAC) and colorectal cancer (CRC) is approximately 10%. In this single-center study, we present 37 patients with PDAC and 48 patients with CRC who were presented at MTB and tested using the large combined DNA/RNA sequencing panel. Content of tumor cells and variant allele frequency scores were evaluated in all tested patients. In case of suspicion of PGV and no previous genetic testing based on the standard guidelines, genetic counseling was recommended regardless of age, sex, and family history. In the PDAC subgroup, five patients were recommended by MTB for genetic counseling based on suspicious genetic findings. Based on a medical geneticist’s decision, germline DNA sequencing was performed in four of these cases, and all of them tested positive for PGV in the following genes: *ATM, ATM, BRCA1*, and *BRCA2*. In the CRC subgroup, no PGV was confirmed in the two patients genetically tested based on the MTB recommendations. Furthermore, we present data from our center’s registry of patients with PDAC and CRC who underwent genetic counseling and germline DNA testing based on the standard screening criteria. Our data confirm that comprehensive genomic profiling of tumor tissue can identify patients with hereditary forms of PDAC, who could remain unidentified by standard screening for hereditary forms of cancer.

## Introduction

Incidence of pancreatic ductal adenocarcinoma (PDAC) is increasing, particularly in western countries. In 2018, 458 918 new cases of PDAC were diagnosed worldwide [1]. In the USA, PDAC is the third most common cause of cancer mortality [2]. Only 15–20% of patients are diagnosed in the early stage of the disease when radical resection is considered the only potentially curative approach [3]. Adjuvant chemotherapy with 5-fluorouracil, oxaliplatin, and irinotecan has improved 5-year survival rate and median overall survival (mOS) to 43.2% and 54.7 months, respectively [4]. However, due to the high rate of adverse events, less intensive chemotherapy is usually considered in daily practice. Thus, the 5-year survival rate in resected PDAC population outside of clinical trials is only 17–20% [3,5,6]. Moreover, approximately 70–80% of patients with PDAC are diagnosed in inoperable locally advanced or metastatic stage, particularly due to the absence of specific symptoms. This results in poor prognosis with mOS of 12–16 months [7,8].

Similar to PDAC, colorectal cancer (CRC) is significantly more frequent in developed countries with almost two million new CRC cases diagnosed globally in 2020 [9]. In the USA, CRC is the second most common cause of cancer-related death [10]. Despite the existence of a CRC screening program in most countries, approximately 25% of newly diagnosed CRC patients have metastatic disease [11]. Moreover, a further 20% of curatively resected patients will develop metachronous metastases [12]. The 5-year relative survival rate for CRC is 65%, regardless of clinical stage [13]. In patients with distant metastases, 5-year survival rate is only 15.1%, despite systemic therapy [13].

New therapeutic options improving prognosis of patients with these advanced tumours are needed. In the last two decades, there has been extensive research in the field of molecular biology; particularly focussing on diagnostic, prognostic, and predictive biomarkers. This has led to a deeper understanding of carcinogenesis, as well as to an expansion of therapeutic options in a wide spectrum of tumour types, including PDAC and CRC.

Performing comprehensive genomic profiling (CGP) of tumour tissue based on next-generation sequencing (NGS) can identify targetable genomic alterations, including homologous recombination deficiency (HRD)-associated variants. Currently, somatic NGS testing on sufficiently large gene panels is considered a standard of care in patients with advanced cancers. In the Czech Republic, NGS testing revealing potential targetable somatic variants is fully covered by health care insurance providers. HRD and non-HRD pathway gene variants may be used for the selection of patients feasible for the biomarker-driven therapy. Heeke *et al*. reported that, in 52 426 NGS tests of tumour tissue, HRD pathway gene variants were detected in 15.4% of PDAC and 15% of CRC [14]. Tumour-only sequencing cannot easily distinguish the somatic and germline origin of these variants. Nevertheless, hereditary factors play an important role in carcinogenesis. In both PDAC and CRC patients, the prevalence of pathogenic germline variants (PGVs) is assumed to be at least 10% [15,16]. Thus, the genetic counselling and identification of these PGVs may have implications on screening of affected population as well as on indication of targeted therapy. However, this prevalence may be underestimated. Up to 57% of PDAC patients harbouring a PGV did not have a suspicious family history and did not meet prior National Comprehensive Cancer Network (NCCN) screening criteria for *BRCA1/2* and *PALB2* germline testing [17]. Another reason may be the use of relatively insufficiently large gene panels [16]. Uson *et al.* demonstrated that universal multigene panel testing (83 genes) in CRC patients indicated regardless of family history or age can detect PGVs in almost 16% of tested patients. Nevertheless, more than 50% of these PGVs would not be detected by using standard guidelines (NCCN, National Society of Genetic Counsellors, and American College of Medical Genetics, 2018 and 2020) or a guideline-specific gene panel [16]. Similarly, Samadder *et al*. conducted prospective multicentre study among patients with multiple solid tumours, including PDAC and CRC. This study demonstrated that universal multigene panel testing (83 genes) may detect PGV in 12.5% of patients (regardless of cancer type, sex, family history of cancer, age at diagnosis, stage of disease, etc.). In half of these patients, PGVs would not have been detected using a standard guideline-based approach [18]. It is necessary to identify methods that may increase the detection of PGVs. One of the possible approaches is the evaluation of the variant allele frequency (VAF). It is defined as the number of variant reads divided by the number of total reads (reported in percentage) within somatic sequencing and may serve as an indicator for germline testing outside the current indication criteria.

In this single-centre study, we evaluate patients with advanced and metastatic tumours who underwent somatic panel testing at the University Hospital Brno, Czech Republic. In PDAC and CRC patients, we describe their clinical characteristics and the prevalence of gene variants suspected to be PGVs (cohort A). In addition to this, we present results from the local genetic database of patients diagnosed with PDAC and CRC who underwent germline sequencing according to standard screening criteria (cohort B).

## Methods

A total of 358 patients with different advanced and pre-treated solid tumours having limited further therapeutic options were indicated for predictive testing of tumour tissue by the molecular tumour board (MTB) at University Hospital Brno, Czech Republic (cohort A). Samples were provided as formalin-fixed paraffin-embedded (FFPE) tissue specimens containing >20% neoplastic cells. Written consent was obtained prior to sample testing.

Molecular genetic predictive testing was performed by the method of combined massively parallel sequencing (NGS) of genomic DNA fragments and total RNA obtained from FFPE tissue sections using the NGS assay TruSight Oncology 500 (Illumina) with the NextSeq 550 System (Illumina). The test is intended for the targeted sequence analysis of 523 cancer-relevant genes (single-nucleotide variants, small insertions/deletions, and copy number variations) and 55 genes for known and novel gene fusions based on the principle of capturing and target enrichment.

Analysis of sequence variants, including copy number variation (CNV), microsatellite instability status (MSI), and tumour mutational burden, was performed using the Clinical Genomics Workspace (CGW, PierianDx, USA) diagnostic software, according to the reference genome GRCh37.p13 annotating NCBI RefSeq v105 reference sequence. Method sensitivity was set at the 5% limit of variant detection in the examined material. The detected sequence variants were identified by CGW according to the currently valid international databases ExAC, dbNSFP, NHLBI ESP, ClinVar, COSMIC, dbSNP, gnomAD, *in silico* prediction algorithms, and therapeutic guidelines.

Based on the NGS analysis, results-targeted therapy was proposed within the MTB. Patients with PDAC and CRC were selected for further analysis. Pancreatic neuroendocrine tumours were not included. If the level of allelic frequency of clinically significant variant (VAF) within tested genes was found to be suspected of the germline variant form, additional genetic testing was recommended by a clinical geneticist attending the MTB in case it was not previously performed according to the standard screening criteria in both diagnostic subgroups (cohort A). Additional written consent from all tested patients was also required.

Germline NGS testing of patients with PDAC and CRC who underwent germline sequencing according to the standard screening criteria (cohort B) was performed by use of in-house BRONCO custom sequencing panel intended for the targeted sequence analysis of 296 genes associated with hereditary tumour predispositions (single-nucleotide variants, small insertions/deletions, and CNV) in combination with digital multiplex ligation-dependent probe amplification (dMLPA) by use of D001 Hereditary Cancer Panel 1 probemix assay (MRC Holland) enabling detection of copy number variants (large exon deletions and duplications) within 29 genes associated with hereditary tumour predispositions. Pathogenic variants were assessed by clinical relevance of affected genes: high risk (relative risk RR > 5), intermediate risk (RR = 2‒5), and low risk (RR = 1‒2 or uncertain).

## Results

Between February 2021 and October 2023, 358 patients (cohort A) with different inoperable, advanced, and pre-treated solid tumours were presented at the MTB and indicated for somatic testing by NGS. Tissue samples suitable for NGS testing were available in 323 cases (90%). Patients with PDAC (*N* = 37, 11.5%) and CRC (*N* = 48, 14.9%) were among the most commonly tested diagnosis, and their median age was 63.5 years at the time of NGS testing. Forty-seven (55.3%) of them were males. The baseline characteristics of these patients are detailed in Table 1.

**Table 1.** Baseline characteristics of patients with PDAC and CRC from Cohort A.

All	*N* = 85
Cancer types	
Pancreatic cancer	37 (11.5%)
Colorectal cancer	48 (14.9%)
**Sex in pancreatic cancer subgroup**	
Male	17 (45.9%)
Female	20 (54.1%)
**Sex in colorectal cancer subgroup**	
Male	30 (62.5%)
Female	18 (37.5%)
**Age at the time of diagnosis, pancreatic cancer subgroup, years**	
Mean	58.3
Median	60.6
Range	34.7–78.7
**Age at the time of diagnosis, colorectal cancer subgroup, years**	
Mean	58.3
Median	60.5
Range	33.9-78.3
**Race**	
White	85 (100%)
**Stage**	
Metastatic	76 (89.4%)
Locally advanced – inoperable	9 (10.6%)
**No. of lines of systemic treatment at the time of NGS testing**	
Median	2
Range	1–5

Based on the content of tumour cells (CTC) in selected tumour areas and the VAF score of clinically significant variant, MTB recommended genetic counselling in five (13.5%) patients with PDAC and in three (6.3%) patients with CRC who had not previously undergone genetic testing according to standard screening criteria because the patient had not been referred for testing by the treating physician. A positive family history of tumours associated with the pathogenic gene variant in first- and second-degree relatives was found in six of them (75%). In one PDAC patient with an *RET* mutation (c.2372A>T, VAF 49.0%, CTC: 30%), MTB recommendation for genetic counselling was not followed by the patient’s physician. Additional germinal testing confirmed a hereditary syndrome in all tested PDAC patients (4/4). Germline variants were found in the following genes: *ATM* (c.3154-2A>G, VAF: 63.1%, CTC: 20%)*, ATM* (c.7630-2A>C, VAF: 58,6%, CTC: 30%), *BRCA1* (c.2762delA, VAF: 54.9%, CTC: 30%), and *BRCA2* (c.2251dupA, VAF: 67.4%, CTC: 20%).

**Figure 1.**
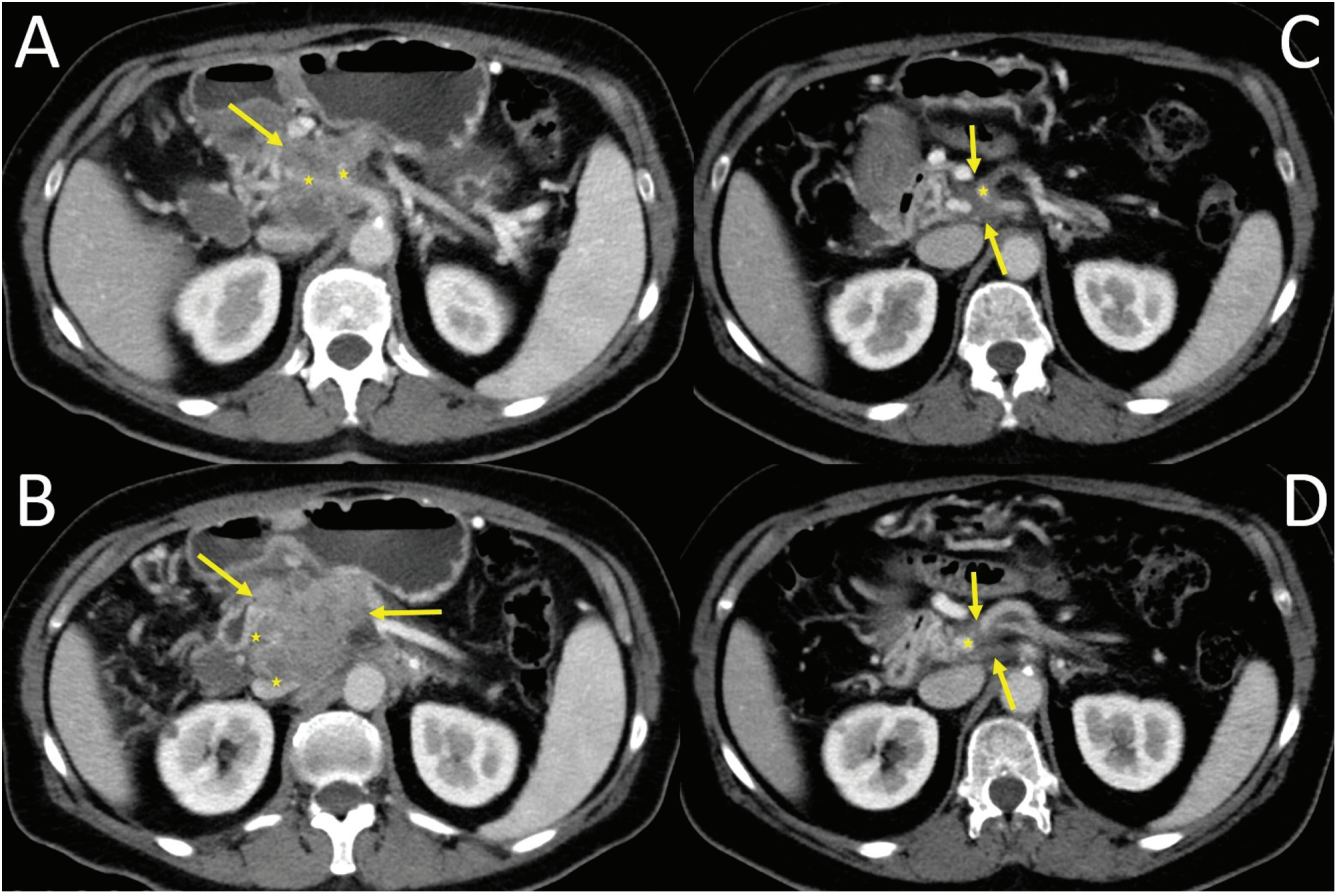
A case of a patient with PDAC with confirmed *BRCA2* mutation. CT in porto-venous phase, axial planes. Images A and B show a large pancreatic tumour (arrows) before treatment (classified as cT4N1M1), which infiltrates the branches of the ceoliac trunk (asterixis), inferior vena cava (asterixis) and completely thromboses the portal vein (asterixis). After treatment (C, D), the original pancreatic infiltrate was significantly reduced in size (arrows; classified as ycT4N0M0, but CT cannot reliably distinguish residual tumour changes from post-treatment changes), pathologic densities continue to contact the branches of the coeliac trunk (asterixis) and stenotize the portal vein (asterixis).

The case of a patient with a confirmed *BRCA2* mutation can be used as an example of the significant impact CGP can have on therapeutic planning. This patient was diagnosed with metastatic pancreatic cancer in September 2022. Baseline staging was determined to be T4 (tumour involves vascular structures), N1 (metastases in 1–3 regional lymph nodes), and M1 (peritoneal distant metastasis) according to the computed tomography (CT) scan. Palliative chemotherapy with gemcitabine and nab-paclitaxel was started immediately after histological confirmation. In parallel with systemic therapy, CGP was performed and a pathogenic gene variant in *BRCA2* was identified. This result led to a modification of systemic therapy to cisplatin-based chemotherapy in November 2022 (gemcitabine plus nab-paclitaxel to gemcitabine plus cisplatin), yet without knowing the definitive result of the germline DNA sequencing. After three months of cisplatin-based therapy, significant tumour shrinkage was observed on the CT scan reassessment (Fig. 1). However, persistent infiltration of vascular structures and T4N0M0 disease was still reported. Cisplatin-based chemotherapy was followed by poly (ADP-ribose) polymerase inhibitor olaparib as a maintenance strategy in February 2023 and radiotherapy (14 fractions of 2.67 Grays over 3 weeks) in April 2023. Olaparib was taken during radiotherapy and onwards until surgical resection with vein replacement performed in June 2023. Surprisingly, a complete pathological response was reported (ypT0N0) by an experienced pathologist with no evidence of metastatic disease perioperatively. At 9 months after resection, the patient is still free of the disease without any subsequent therapy. In addition to the excellent therapeutic response achieved due to the finding of the *BRCA2* mutation in the somatic tumour DNA and the respective change of therapeutic regimen, this finding also allowed subsequent confirmation of its germline origin and genetic counselling for the patient and her family.

For the CRC subgroup, only three patients were tested for a suspicion of germline variants in *FANCL* (c.31C>T, VAF: 41.9%, CTC: 40%), *APC* (c.1548 + 1G>T, VAF: 78.8%, CTC: 30%), and *FANCG* (c.313G>6, VAF: 53.4%, CTC: 30%) genes; however, no hereditary syndrome was confirmed in the first two cases. In a patient with *FANCG* variant, the final genetic report is not still available. A summary of PDAC and CRC patients with a suspicion of PGV according to the somatic testing is detailed in Table 2.

**Table 2.** Patients with PDAC and CRC from Cohort A referred for germline testing of suspected genes based on tissue sequencing and MTB recommendation.

Diagnosis	Sex	Age (years)	Suspected gene and variant—somatic testing	VAF score (%)	Content of tumour cells in selected tumour area (%)	Recommendation for the genetic counselling followed by a physician YES/NO	Hereditary syndrome confirmed YES/NO	Family history positive YES/NO	Clinical outcomes YES/NO
PDAC	M	53	*ATM* (NM_000051.3) c.3154-2A>G p.?	63.1	20	YES	YES	NO	NO
PDAC	F	59	*BRCA2* (NM_000059.3) c.2251dupA p.T751Nfs*2	67.4	20	YES	YES	YES	YES—pCR
PDAC	M	62	*RET* (NM_020975.4) c.2372A>T p.Y791F	49.0	30	NO	NA	YES	NA
PDAC	M	43	*BRCA1* (NM_007300.3) c.2762delA p.Q921Rfs*79	54.9	30	YES	YES	YES	YES—cCR
PDAC	F	75	*ATM* (NM_000051.3) c.7630-2A>C p.?	58.6	30	YES	YES	YES	NO
CRC	M	65	*FANCL* (NM_001114636.1) c.31C>T p.Q11*	41.9	40	YES	NO	YES	NO
CRC	M	66	*APC* (NM_000038.5) c.1548 + 1G>T p.?	78.8	30	YES	NO	NO	NO
CRC	F	58	*FANCG* (NM_004629.1) c.313G>6 p.E105*	53.4	30	YES	NA	YES	NA

cCR, clinical complete remission (not resected); CRC, colorectal cancer; F, female; M, male; NA, not available; pCR, pathologic complete remission (resected); PDAC, pancreatic ductal adenocarcinoma; VAF, variant allele frequency.

Cohort B includes PDAC and CRC patients who were consulted by a clinical geneticist and tested for germline variants between January 2018 and November 2023. Indication for testing was based on the screening criteria that was valid at the time the patient was tested regardless of the MTB recommendation. Germline testing was performed by NGS and dMLPA. The median age of patients in both subgroups was 59 years at the time of genetic counselling. In the PDAC subgroup, 50 patients (26 females and 24 males) were analysed with 12 (24.0%) patients diagnosed with a PGV (12.0% high-risk variants, 10.0% intermediate- and low-risk variants). A further 12 (24.0%) patients were diagnosed with a variant of uncertain significance (VUS) only (Table 3). In the CRC subgroup of patients, 83 patients (42 females and 41 males) underwent germline testing with 14 (16.9%) tested positively for PGV (8.3% high-risk variants, 8.3% intermediate- and low-risk variants). In the 22 following patients (26.5%), only VUS was detected (Table 4).

**Table 3.** Patients with PDAC from Cohort B who were consulted by a clinical geneticist and tested for germline mutations based on screening criteria regardless of MTB recommendations.

Sex	Age (years)	Gene	Gene variant	High-risk variant YES/NO	Intermediate- and low-risk variant YES/NO	VUS YES/NO
F	46	*MUTYH*	c.536A>G	NO	YES	NO
F	45	*ERCC2*	c.1867dup	NO	YES	NO
M	39	*MUTYH* *MSH3*	c.453_458dup c.2732T>G	NO NO	YES NO	NO YES
F	79	*BRCA2*	c.1023_1024del	YES	NO	NO
F	72	*MPL*	c.127C>T	NO	YES	NO
F	68	*POLE*	c.4523G>A	NO	NO	YES
M	47	*BRCA2*	c.8338G>A	NO	NO	YES
F	66	*PMS1* *MUTYH*	c.654dup c.1301C>T	YES NO	NO NO	NO YES
F	40	*BRCA2* *CHEK2*	c.9435_9436 c.1421G>A	YES YES	NO NO	NO NO
F	70	*MLH3*	c.1390T>C	NO	NO	YES
M	53	*ATM*	c.3154-2A>G	YES	NO	NO
F	59	*BRCA2*	c.2251dup	YES	NO	NO
F	70	*STK11*	c.1150C>T	NO	NO	YES
M	52	*PMS2*	c.113C>T	YES	NO	NO
M	42	*BRCA1*	c.2762del	YES	NO	NO
M	76	*POLE*	c.861T>A	NO	NO	YES
M	68	*MLH3*	c.562C>T	NO	NO	YES
F	43	*PALB2*	c.1544A>G	NO	NO	YES
F	60	*NF1*	c.7781G>T	NO	NO	YES
M	61	*MSH3*	c.2336G>A	NO	NO	YES
F	74	*ATM*	c.7630-2A>C	YES	NO	NO
M	60	*MLH3* *POLD1*	c.1724A>G) c.961G>A	NO NO	NO NO	YES YES
F	59	*PALB2*	c.3235G>T	NO	NO	YES
M	64	*ATM*	c.5218A>G	NO	NO	YES

F, female; M, male; VUS, variant of uncertain significance.

**Table 4.** Patients with CRC from Cohort B who were consulted by a clinical geneticist and tested for germline mutations based on screening criteria regardless of MTB recommendations.

Sex	Age (years)	Gene	Gene variant	High-risk variant YES/NO	Intermediate- and low-risk variant YES/NO	VUS YES/NO
M	41	*POLD1* *MSH3*	c.154_171 del18 c.196_204 del9	NO NO	NO NO	YES YES
M	69	*PMS1* *POLD1*	c.479C>T c.1294C>G	YES NO	NO NO	NO YES
M	79	*SMAD4*	c.554C>T	NO	NO	YES
F	53	*BRCA1*	c.213-12A>G	YES	NO	NO
F	53	*ERCC2*	c.361-1G>A	NO	YES	NO
M	67	*PMS1*	c.224C>T	NO	NO	YES
M	48	*SMAD4*	c.10_11delAT	YES	NO	NO
F	68	*BRIP1*	c.728T>C	NO	NO	YES
M	45	*MLH1*	c.1990-2A>C	YES	NO	NO
F	40	*MLH3* *PMS1*	c.2115_2118del c.2380A>T	NO NO	YES YES	NO NO
M	49	*POLD1*	c.455C>T	NO	NO	YES
M	68	*ATM*	c.3279_3282del (p.Asn1094fs)	YES	NO	NO
F	40	*POLD1*	c.328C>T	NO	NO	YES
F	48	*BRCA1*	c.878C>T	NO	NO	YES
M	81	*POLE*	c.6019G>A	NO	NO	YES
M	60	*ATM* *POLE* *MSH6*	c.7322T>C c.1583C>T c.1061C>T	NO NO NO	NO NO NO	YES YES YES
M	67	*POLE*	c.5650A>G	NO	NO	YES
M	43	*APC*	c.7105C>T	NO	NO	YES
M	62	*FAN1*	c.2916 + 2T>G	NO	YES	NO
F	73	*MSH2*	c.2255G>A	NO	NO	YES
M	44	*MSH2*	c.131del	YES	NO	NO
M	58	*MSH5* *BRCA2*	c.404G>A c.-12T>C	NO NO	NO NO	YES YES
M	64	*POLE*	c.6019G>A	NO	NO	YES
F	43	*PMS2* *BARD1*	c.1567T>A c.2224T>A	NO NO	NO NO	YES YES
F	72	*CDK12* *BLM*	c.1047-2A>G c.44G>A	NO NO	YES NO	NO YES
M	73	*CDKN2B*	c.256G>A	NO	NO	YES
F	54	*PMS2*	c.2240G>C	NO	NO	YES
F	54	*PMS1*	c.1912G>A	NO	NO	YES
F	42	*PALB2*	c.1544A>G	NO	NO	YES
F	35	*RECQL5*	c.717T>G	NO	YES	NO
F	50	*APEX1*	c.872dup	NO	YES	NO
M	34	*APC* *MITF* *MPL* *CHEK1*	exon 15-16 deletion (hg19) c.952G>A c.992G>A c.236G>A	YES NO NO NO	NO YES YES YES	NO NO NO NO
F	59	*TP53*	c.1016A>G	NO	NO	YES
F	48	*MSH6*	c.3600A>G	NO	NO	YES
M	80	*MSH5*	c.2419A>G	NO	NO	YES
M	49	*CHEK2* *BRCA2*	exon 9-10 deletion c.1793C>T	YES NO	NO NO	NO YES
M	72	*NBN*	c.171 + 1G>C	NO	YES	NO

F, female; M, male; VUS, variant of uncertain significance.

## Discussion

This single-centre retrospective analysis highlights the clinical utility of CGP with regards to the possible detection of PGVs among patients with advanced PDAC and CRC with no previous genetic counselling. All patients were treated at the University Hospital Brno, Czech Republic. In total, 37 PDAC and 48 CRC patients were tested. The main output of somatic testing is to determine the prediction of the effectiveness of targeted therapy considering the available evidence. In PDAC, actionable gene variants are present in up to 25% of cases [19]. In CRC, target therapy may be indicated in up to 80% of patients [20]. However, somatic testing and assessment of the VAF score of clinically significant variants in combination with the CTC in selected tumour areas may indicate a suspected hereditary syndrome.

While germline testing should be currently indicated for all patients with PDAC regardless of the clinical stage, sex, age, and family history, not all physicians follow this recommendation in their daily routine [21,22]. Sequencing of tumour tissue identifying targetable gene variants is indicated in all advanced and metastatic PDAC. In the Czech Republic, somatic sequencing is fully reimbursed by all seven public health care insurance providers. Although the number of PDAC patients who underwent somatic testing at our centre was low, we identified five patients with no previous genetic counselling with suspicion of PGV. The recommendation of MTB for germline testing was followed in four (80%) of them, and all were diagnosed with a hereditary syndrome (PGV in genes *ATM, ATM, BRCA1, BRCA2*). A family history of the patient with PGV in *ATM* (NM_000051.3) was negative, and this variant has developed *de novo*. In the patient with the *BRCA2* variant, systemic chemotherapy was modified to a platinum-based regimen according to the results of somatic testing. This modification resulted in complete pathologic remission despite the original metastatic disease. Another PDAC patient, who relapsed (multiple liver metastases) during adjuvant chemotherapy with gemcitabine and subsequently underwent somatic testing with a suspicion of a germline *BRCA1* variant, reached a complete clinical remission after platinum-based palliative systemic therapy. A germline *BRCA1* variant was finally confirmed during palliative therapy.

In CRC patients, germline sequencing should be recommended if screening criteria were met (if there are at least three relatives in the family with carcinoma associated with hereditary nonpolyposis colorectal cancer—Lynch syndrome, endometrial carcinoma, carcinoma of small intestine, ureter, and kidney; if one of them is a first-degree relative of the other two; if at least two generations are affected; if at least one patient was younger than 50 years at the time of diagnosis) [23]. Similar to PDAC, CRC patients with proven advanced or metastatic disease should be considered for somatic sequencing. The MTB recommended only three CRC patients with no previous genetic counselling for germline testing at our centre. However, no hereditary syndrome was confirmed in two tested patients.

Currently, an increasing number of trials have demonstrated improved survival parameters and quality of life if actionable gene variants were targeted by a specific inhibitor. In the following text, we mention the most relevant gene variants with the possibility of targeted therapy for both diagnostic subgroups. Kirsten rat sarcoma viral oncogene homolog (*KRAS*) has been the most studied oncogene that has the highest mutation rate in the vast majority of tumour types, including PDAC (>90%) and CRC (~50%). Until recently, *KRAS* was historically considered undruggable for decades. However, *KRAS* G12C-mutant selective irreversible inhibitors, such as sotorasib and adagrasib, have demonstrated meaningful clinical activity in heavily pre-treated patients with metastatic *KRAS* G12C-mutated PDAC and CRC [24,25]. Many other *KRAS* selective inhibitors are currently being investigated in ongoing clinical trials, including multi-KRAS p.G12X inhibitors (*KRAS* p.G12A, *KRAS* p.G12D, *KRAS* p.G12R, *KRAS* p.G12S, or *KRAS* p.G12V) [26]. In 10% of PDAC patients, no *KRAS* mutations are found. In this *KRAS* wild-type population, multiple alternative gene variants are commonly presented and may be targeted by specific inhibitors.

Human epidermal growth factor receptor 2 (*HER2*) amplification is present in approximately 2% and 2–5% of PDAC and CRC patients, respectively [27,28]. Multiple *HER2* inhibitors improved mOS and median progression-free survival (PFS) in metastatic CRC [29,30]. However, only a marginal effect was observed in PDAC patients [31,32].

Activating p.V600E mutations in *B-raf* murine sarcoma viral oncogene homolog B (*BRAF*) are presented in 3% and 10% of PDAC and CRC, respectively [33,34]. Inhibition of *BRAF* may lead to reactivation of MAPK signalling, including *EGFR* and *MEK*, which are considered to be a dominant driver in many tumour types [35]. A combination of *BRAF* p.V600E and *MEK* inhibitors has demonstrated significantly improved survival parameters in a wide spectrum of tumour types [36]. Based on the ROAR basket trial results, a combination of dabrafenib and trametinib should be considered in all tumours harbouring *BRAF* p.V600E mutation, including PDAC [36,37]. In CRC, a combination of *BRAF* p.V600E inhibitor encorafenib, *EGFR* inhibitor cetuximab, and *MEK* inhibitor binimetinib resulted in significantly longer mOS and higher overall response rate (ORR) compared to standard therapy [38].

*BRCA1/2* are genes encoding proteins with a key role in homologous recombination [39,40]. Mutations and loss-of-function variants of these genes are responsible for HRD, which is associated with the inability to repair double-strand DNA breaks. Thus, PDAC and other tumours harbouring an HRD are significantly responding to DNA damaging and cross-linking agents such as platinum derivatives, as was demonstrated in our two cases [41,42]. The efficacy of platinum-based chemotherapy among PDAC patients with germline variants in HRD-associated genes was confirmed in multiple studies with a response rate of 70% [43,44]. Somatic *BRCA1/2* variants are present in 2% of PDAC. Rucaparib is a PARP inhibitor, and its effect has been tested in pre-treated patients with either somatic or germline *BRCA1/2* variants. One partial response and one complete response were confirmed in a group of advanced or metastatic PDAC with somatic variants [45]. In CRC patients with variants in HRD-associated genes, only a marginal effect of platinum-based therapy was demonstrated, and further studies are needed [46].

*NTRK1/2/3* fusions are rare in PDAC and CRC with a prevalence of less than 0.8% [47,48]. However, inhibitors such as larotrectinib and entrectinib are highly effective as monotherapy among patients with *TRK*-fusion cancers, including PDAC and CRC. Larotrectinib led to an ORR of 79% with 16% CR and a median duration of responses of 35.2 months [49]. Currently, *TRK* inhibitors are approved by the FDA and EMA as a tumour-agnostic therapy.

Microsatellite instability (MSI-H)/mismatch repair deficient (dMMR) is present in less than 2% of PDAC and in 15% of CRC patients and is associated with a deficiency in protein products of *MSH2, MLH1*, *MSH6,* or *PMS2* genes [50–52]. Lynch syndrome is caused by inherited germline variants in one allele followed by somatic inactivation of the wild-type allele in a colonic epithelial cell. The second cause of MSI-H/dMMR is somatic inactivation of both of the alleles [52]. Genetic counselling should be recommended in all cancer patients with MSI-H, regardless of age [53]. In PDAC, MSI-H/dMMR is commonly associated with wild-type *KRAS* and *TP53* [50]. Somatic or germline mutations in mentioned genes may result in response to immunotherapy with checkpoint inhibitors. Pembrolizumab is a PD-1 inhibitor and leads to significantly longer PFS in first-line therapy for MSI-H/dMMR metastatic CRC compared to standard chemotherapy [54,55]. However, in MSI-H/dMMR patients with PDAC, only one complete and three partial responses were observed among 22 analysed patients in a single-arm phase II trial KEYNOTE-158 [56].

Although the number of PDAC and CRC patients who underwent CGP for therapeutic planning at our centre is small, results suggest that this approach may also help to detect hereditary syndromes, especially in PDAC. It may have significant outcomes particularly among patients who did not have a suspicious family history and did not meet standard screening criteria for germline testing.

## References

[1] Rawla P , Sunkara T, Gaduputi V. Epidemiology of pancreatic cancer: global trends, etiology and risk factors. World J Oncol 2019;10:10–27. https://doi.org/10.14740/wjon1166 30834048 PMC6396775

[2] Siegel RL , Miller KD, Fuchs HE, et al Cancer statistics, 2021. CA Cancer J Clin 2021;71:7–33. https://doi.org/10.3322/caac.21654 33433946

[3] Kardosh A , Lichtensztajn DY, Gubens MA, et al Long-term survivors of pancreatic cancer. Pancreas 2018;47:958–66. https://doi.org/10.1097/MPA.0000000000001133 30074526 PMC6095724

[4] Conroy T , Castan F, Lopez A, et al; Canadian Cancer Trials Group and the Unicancer-GI–PRODIGE Group. Five-year outcomes of FOLFIRINOX vs gemcitabine as adjuvant therapy for pancreatic cancer: a randomized clinical trial. JAMA Oncol 2022;8:1571–8. https://doi.org/10.1001/jamaoncol.2022.3829 36048453 PMC9437831

[5] Strobel O , Lorenz P, Hinz U, et al Actual five-year survival after upfront resection for pancreatic ductal adenocarcinoma: Who beats the odds? Ann Surg 2022;275:962–71. https://doi.org/10.1097/SLA.0000000000004147 32649469

[6] Versteijne E , van Dam JL, Suker M, et al; Dutch Pancreatic Cancer Group. Neoadjuvant chemoradiotherapy versus upfront surgery for resectable and borderline resectable pancreatic cancer: long-term results of the dutch randomized PREOPANC trial. J Clin Oncol 2022;40:1220–30. https://doi.org/10.1200/JCO.21.02233 35084987

[7] Conroy T , Desseigne F, Ychou M, et al; Groupe Tumeurs Digestives of Unicancer. FOLFIRINOX versus gemcitabine for metastatic pancreatic cancer. N Engl J Med 2011;364:1817–25. https://doi.org/10.1056/NEJMoa1011923 21561347

[8] van Veldhuisen E , van den Oord C, Brada LJ, et al; Dutch Pancreatic Cancer Group and International Collaborative Group on Locally Advanced Pancreatic Cancer. Locally advanced pancreatic cancer: work-up, staging, and local intervention strategies. Cancers (Basel). 2019;11:976. https://doi.org/10.3390/cancers11070976 31336859 PMC6679311

[9] Morgan E , Arnold M, Gini A, et al Global burden of colorectal cancer in 2020 and 2040: incidence and mortality estimates from GLOBOCAN. Gut 2023;72:338–44. https://doi.org/10.1136/gutjnl-2022-327736 36604116

[10] Siegel RL , Wagle NS, Cercek A, et al Colorectal cancer statistics, 2023. CA Cancer J Clin 2023;73:233–54. https://doi.org/10.3322/caac.21772 36856579

[11] Hernandez Dominguez O , Yilmaz S, Steele SR. Stage IV colorectal cancer management and treatment. J Clin Med 2023;12:2072. https://doi.org/10.3390/jcm12052072 36902858 PMC10004676

[12] Väyrynen V , Wirta EV, Seppälä T, et al Incidence and management of patients with colorectal cancer and synchronous and metachronous colorectal metastases: a population-based study. BJS Open 2020;4:685–92. https://doi.org/10.1002/bjs5.50299 32543788 PMC7397359

[13] Cancer of the Colon and Rectum - Cancer Stat Facts. SEER. https://seer.cancer.gov/statfacts/html/colorect.html (19 October 2023, date last accessed).

[14] Heeke AL , Pishvaian MJ, Lynce F, et al Prevalence of homologous recombination–related gene mutations across multiple cancer types. JCO Precis Oncol 2018;2:1–13. https://doi.org/10.1200/PO.17.00286 PMC6139373 30234181

[15] Dudley B , Karloski E, Monzon FA, et al Germline mutation prevalence in individuals with pancreatic cancer and a history of previous malignancy. Cancer 2018;124:1691–700. https://doi.org/10.1002/cncr.31242 29360161

[16] Uson PLS , Riegert-Johnson D, Boardman L, et al Germline cancer susceptibility gene testing in unselected patients with colorectal adenocarcinoma: a multicenter prospective study. Clin Gastroenterol Hepatol 2022;20:e508–28. https://doi.org/10.1016/j.cgh.2021.04.013 33857637

[17] Holter S , Borgida A, Dodd A, et al Germline BRCA mutations in a large clinic-based cohort of patients with pancreatic adenocarcinoma. J Clin Oncol 2015;33:3124–9. https://doi.org/10.1200/JCO.2014.59.7401 25940717

[18] Samadder NJ , Riegert-Johnson D, Boardman L, et al Comparison of universal genetic testing vs guideline-directed targeted testing for patients with hereditary cancer syndrome. JAMA Oncol 2021;7:230–7. https://doi.org/10.1001/jamaoncol.2020.6252 33126242 PMC7600058

[19] Pishvaian MJ , Bender RJ, Halverson D, et al Molecular profiling of patients with pancreatic cancer: initial results from the know your tumor initiative. Clin Cancer Res 2018;24:5018–27. https://doi.org/10.1158/1078-0432.CCR-18-0531 29954777

[20] Yekedüz E , Akbulut H, Utkan G, et al Genomic landscape of actionable mutations in primary and metastatic tissues of colon adenocarcinoma. Cureus 2022;14:e24175. https://doi.org/10.7759/cureus.24175 35592200 PMC9110093

[21] Tempero MA , Malafa MP, Al-Hawary M, et al Pancreatic adenocarcinoma, version 2.2021, NCCN clinical practice guidelines in oncology. J Natl Compr Canc Netw 2021;19:439–57. https://doi.org/10.6004/jnccn.2021.0017 33845462

[22] Kurian AW , Abrahamse P, Furgal A, et al Germline genetic testing after cancer diagnosis. JAMA 2023;330:43–51. https://doi.org/10.1001/jama.2023.9526 37276540 PMC10242510

[23] Vasen HF , Watson P, Mecklin JP, et al New clinical criteria for hereditary nonpolyposis colorectal cancer (HNPCC, Lynch syndrome) proposed by the International Collaborative group on HNPCC. Gastroenterology 1999;116:1453–6. https://doi.org/10.1016/s0016-5085(99)70510-x 10348829

[24] Strickler JH , Satake H, George TJ, et al Sotorasib in KRAS p.G12C-Mutated advanced pancreatic cancer. N Engl J Med 2023;388:33–43. https://doi.org/10.1056/NEJMoa2208470 36546651 PMC10506456

[25] Yaeger R , Weiss J, Pelster MS, et al Adagrasib with or without Cetuximab in colorectal cancer with mutated KRAS G12C. N Engl J Med 2023;388:44–54. https://doi.org/10.1056/NEJMoa2212419 36546659 PMC9908297

[26] Koltun ES , Rice MA, Gustafson WC, et al Abstract 3597: direct targeting of KRASG12X mutant cancers with RMC-6236, a first-in-class, RAS-selective, orally bioavailable, tri-complex RASMULTI(ON) inhibitor. Cancer Res 2022;82:3597–3597. https://doi.org/10.1158/1538-7445.AM2022-3597

[27] Tavberidze N , Zhang W. HER2 (ERBB2) alterations in colorectal cancers. Human Pathol Rep 2022;28:300628. https://doi.org/10.1016/j.hpr.2022.300628

[28] Chou A , Waddell N, Cowley MJ, et al Clinical and molecular characterization of HER2 amplified-pancreatic cancer. Genome Med 2013;5:78. https://doi.org/10.1186/gm482 24004612 PMC3978667

[29] Strickler JH , Cercek A, Siena S, et al; MOUNTAINEER investigators. Tucatinib plus trastuzumab for chemotherapy-refractory, HER2-positive, RAS wild-type unresectable or metastatic colorectal cancer (MOUNTAINEER): a multicentre, open-label, phase 2 study. Lancet Oncol 2023;24:496–508. https://doi.org/10.1016/S1470-2045(23)00150-X 37142372

[30] Yoshino T , Di Bartolomeo M, Raghav K, et al; DESTINY-CRC01 investigators. Final results of DESTINY-CRC01 investigating trastuzumab deruxtecan in patients with HER2-expressing metastatic colorectal cancer. Nat Commun 2023;14:3332. https://doi.org/10.1038/s41467-023-38032-4 37286557 PMC10247780

[31] Meric-Bernstam F , Makker V, Oaknin A, et al Efficacy and safety of trastuzumab deruxtecan in patients With HER2-expressing solid tumors: primary results from the DESTINY-PanTumor02 phase II trial. J Clin Oncol 2023;42:47–58. https://doi.org/10.1200/JCO.23.02005 37870536 PMC10730032

[32] Harder J , Ihorst G, Heinemann V, et al Multicentre phase II trial of trastuzumab and capecitabine in patients with HER2 overexpressing metastatic pancreatic cancer. Br J Cancer 2012;106:1033–8. https://doi.org/10.1038/bjc.2012.18 22374460 PMC3304403

[33] Caputo F , Santini C, Bardasi C, et al BRAF-mutated colorectal cancer: clinical and molecular insights. Int J Mol Sci 2019;20:5369. https://doi.org/10.3390/ijms20215369 31661924 PMC6861966

[34] Sasankan S , Rebuck L, Darrah G, et al Metastatic pancreatic cancer with BRAF and P53 mutations: case report of therapeutic response to doublet targeted therapy. Case Rep Oncol 2020;13:1239–43. https://doi.org/10.1159/000510096 33250737 PMC7670384

[35] Tian J , Chen JH, Chao SX, et al Combined PD-1, BRAF and MEK inhibition in BRAFV600E colorectal cancer: a phase 2 trial. Nat Med 2023;29:458–66. https://doi.org/10.1038/s41591-022-02181-8 36702949 PMC9941044

[36] Subbiah V , Kreitman RJ, Wainberg ZA, et al Dabrafenib plus trametinib in BRAFV600E-mutated rare cancers: the phase 2 ROAR trial. Nat Med 2023;29:1103–12. https://doi.org/10.1038/s41591-023-02321-8 37059834 PMC10202803

[37] Butt SUR , Mejias A, Morelli C, et al BRAF/MEK inhibitors for BRAF V600E-mutant cancers in non-approved setting: a case series. Cancer Chemother Pharmacol 2021;87:437–41. https://doi.org/10.1007/s00280-021-04234-0 33537843

[38] Kopetz S , Grothey A, Yaeger R, et al Encorafenib, binimetinib, and cetuximab in BRAF V600E-mutated colorectal cancer. N Engl J Med 2019;381:1632–43. https://doi.org/10.1056/NEJMoa1908075 31566309

[39] Moynahan ME , Pierce AJ, Jasin M. BRCA2 is required for homology-directed repair of chromosomal breaks. Mol Cell 2001;7:263–72. https://doi.org/10.1016/s1097-2765(01)00174-5 11239455

[40] Moynahan ME , Chiu JW, Koller BH, et al Brca1 controls homology-directed DNA repair. Mol Cell 1999;4:511–8. https://doi.org/10.1016/s1097-2765(00)80202-6 10549283

[41] Turner N , Tutt A, Ashworth A. Hallmarks of ‘BRCAness’ in sporadic cancers. Nat Rev Cancer 2004;4:814–9. https://doi.org/10.1038/nrc1457 15510162

[42] Husain A , He G, Venkatraman ES, et al BRCA1 up-regulation is associated with repair-mediated resistance to cis-diamminedichloroplatinum(II). Cancer Res 1998;58:1120–3. 9515792

[43] O’Reilly EM , Lee JW, Zalupski M, et al Randomized, multicenter, phase II trial of gemcitabine and cisplatin with or without veliparib in patients with pancreas adenocarcinoma and a germline BRCA/PALB2 mutation. J Clin Oncol 2020;38:1378–88. https://doi.org/10.1200/JCO.19.02931 31976786 PMC7193749

[44] Fogelman D , Sugar EA, Oliver G, et al Family history as a marker of platinum sensitivity in pancreatic adenocarcinoma. Cancer Chemother Pharmacol 2015;76:489–98. https://doi.org/10.1007/s00280-015-2788-6 26126726 PMC4811030

[45] Shroff RT , Hendifar A, McWilliams RR, et al Rucaparib monotherapy in patients with pancreatic cancer and a known deleterious BRCA mutation. JCO Precis Oncol 2018;2018:PO.17.00316. https://doi.org/10.1200/PO.17.00316 30051098 PMC6057747

[46] Lee MS , Kopetz S. Are Homologous recombination deficiency mutations relevant in colorectal cancer? JNCI 2021;114:176–8. https://doi.org/10.1093/jnci/djab170 PMC8826393 34469539

[47] Allen MJ , Zhang A, Bavi P, et al Molecular characterisation of pancreatic ductal adenocarcinoma with NTRK fusions and review of the literature. J Clin Pathol 2023;76:158–65. https://doi.org/10.1136/jclinpath-2021-207781 34583947

[48] Wang H , Li Z, Ou Q, et al NTRK fusion positive colorectal cancer is a unique subset of CRC with high TMB and microsatellite instability. Cancer Med 2022;11:2541–9. https://doi.org/10.1002/cam4.4561 35506567 PMC9249987

[49] Bokemeyer C , Vassal G, Italiano A, et al Impact of disease evolution on efficacy outcomes from larotrectinib in patients with locally advanced or metastatic tropomyosin receptor kinase fusion–positive solid tumors. JCO Precis Oncol 2021;5:PO.21.00089. https://doi.org/10.1200/PO.21.00089 34568715 PMC8457788

[50] Luchini C , Brosens LAA, Wood LD, et al Comprehensive characterisation of pancreatic ductal adenocarcinoma with microsatellite instability: histology, molecular pathology and clinical implications. Gut 2021;70:148–56. https://doi.org/10.1136/gutjnl-2020-320726 32350089 PMC7211065

[51] Sinicrope FA , Sargent DJ. Molecular pathways: microsatellite instability in colorectal cancer: prognostic, predictive, and therapeutic implications. Clin Cancer Res 2012;18:1506–12. https://doi.org/10.1158/1078-0432.CCR-11-1469 22302899 PMC3306518

[52] Gian L , Lorena B, Cinzia A, et al Microsatellite instability in colorectal cancer. Acta Biomed 2018;89:97–101. https://doi.org/10.23750/abm.v89i9-S.7960 30561401 PMC6502181

[53] Weiss JM , Gupta S, Burke CA, et al NCCN Guidelines^®^ Insights: Genetic/Familial High-Risk Assessment: Colorectal, Version 1.2021. *J Natl Compr Canc Netw* 2021;19:1122–32. 10.1164/jnccn.2021.0048 34666312

[54] André T , Shiu KK, Kim TW, et al; KEYNOTE-177 Investigators. Pembrolizumab in microsatellite-instability-high advanced colorectal cancer. N Engl J Med 2020;383:2207–18. https://doi.org/10.1056/NEJMoa2017699 33264544

[55] Diaz LA , Shiu KK, Kim TW, et al; KEYNOTE-177 Investigators. Pembrolizumab versus chemotherapy for microsatellite instability-high or mismatch repair-deficient metastatic colorectal cancer (KEYNOTE-177): final analysis of a randomised, open-label, phase 3 study. Lancet Oncol 2022;23:659–70. https://doi.org/10.1016/S1470-2045(22)00197-8 35427471 PMC9533375

[56] Marabelle A , Le DT, Ascierto PA, et al Efficacy of Pembrolizumab in Patients With Noncolorectal High Microsatellite Instability/Mismatch Repair-Deficient Cancer: Results From the Phase II KEYNOTE-158 Study. J Clin Oncol 2020;38:1–10. https://doi.org/10.1200/JCO.19.02105 31682550 PMC8184060

